# Computational Methods for Protein Identification from Mass Spectrometry Data

**DOI:** 10.1371/journal.pcbi.0040012

**Published:** 2008-02-29

**Authors:** Leo McHugh, Jonathan W Arthur

**Affiliations:** National Center for Biotechnology Information, United States of America

## Abstract

Protein identification using mass spectrometry is an indispensable computational tool in the life sciences. A dramatic increase in the use of proteomic strategies to understand the biology of living systems generates an ongoing need for more effective, efficient, and accurate computational methods for protein identification. A wide range of computational methods, each with various implementations, are available to complement different proteomic approaches. A solid knowledge of the range of algorithms available and, more critically, the accuracy and effectiveness of these techniques is essential to ensure as many of the proteins as possible, within any particular experiment, are correctly identified. Here, we undertake a systematic review of the currently available methods and algorithms for interpreting, managing, and analyzing biological data associated with protein identification. We summarize the advances in computational solutions as they have responded to corresponding advances in mass spectrometry hardware. The evolution of scoring algorithms and metrics for automated protein identification are also discussed with a focus on the relative performance of different techniques. We also consider the relative advantages and limitations of different techniques in particular biological contexts. Finally, we present our perspective on future developments in the area of computational protein identification by considering the most recent literature on new and promising approaches to the problem as well as identifying areas yet to be explored and the potential application of methods from other areas of computational biology.

## Introduction

Proteomics is a relatively new but rapidly maturing discipline within life science research for understanding the biology of an organism via the large-scale study of the proteins expressed by the organism. There is already a vast body of literature applying proteomics in many different areas of clinical and biochemical interest and in the study of the pathogenesis, development, prevention, and treatment of a wide range of diseases [1].

Protein identification is a key and essential step in the field of proteomics. The examination of patterns of protein expression alone can, of course, lead to important discoveries, including, for example, classification of samples on the basis of a particular pattern. However, without identifying the proteins known to be critically involved in the system under investigation, it is not possible to delve into the biological explanation for these patterns or to develop hypotheses as to the underlying biology of the system of interest. Thus, while protein identification may often be overlooked or taken for granted, it remains the key initial step in elucidating the biology of an organism by studying its protein expression. Our ability to maximize the benefit of proteomics to life science research is often dependent on our ability to accurately, quickly, and completely identify the full complement of proteins found in our samples of interest.

The exponential growth in DNA sequence and protein databases, coupled with a similar growth in machine throughput, and the critical nature of protein identification to the proteomics process, has seen an explosion in interest in protein identification. For example, both the number and proportion of National Center for Biotechnology (NCBI) articles containing the phrase “protein identification” has seen exponential growth in the past decade.

Mass spectrometry has emerged as the primary tool for protein identification and is the cornerstone of proteomics. While it was first used almost a century ago [2], the use of mass spectrometry for biological applications dates from the 1950s [3], and its use in peptide identification dates from the 1960s [4]. Accuracy, speed, and sample weight range have seen improvements spanning many orders of magnitude in recent decades [5], making mass spectrometry one of the greatest scientific success stories of the twentieth century. No fewer than five Nobel laureates have been awarded the distinction for their pioneering work in mass spectrometry.

The speed and accuracy of these machines make them amenable to the high-throughput applications required not just in proteomics, but also in many other areas of the life sciences, resulting in rapid developments in hardware, software, and data management in the last decade. When we consider the use of mass spectrometers for protein identification, these rapid developments have lead to a bewildering number of instrument configurations, analysis algorithms, and data formats. Mass spectrometry is often critically important in a number of research pipelines. As such, biologists, and computational biologists especially, are often expected to meaningfully manage and interpret mass spectrometry data, requiring an understanding of the most up-to-date methods available to maximize true protein identifications and minimize false identifications for their particular application. This insight into protein identification algorithms is important because often the results may be ambiguous, and the biases chosen to make the problem computationally tractable can radically affect the result.

Despite the improvements in mass spectrometry hardware and the reliability of modern protein identification software, several studies involving a range of mass spectrometers, datasets, and identification algorithms have shown in each case that fewer than half of the proteins in a complex proteomic sample can be identified [6–13]. Given the critical role of protein identification in proteomic analysis, this review aims to explore this apparent upper limit on the effectiveness of current protein identification algorithms and to give relevant background information and practical suggestions to computational biologists and life scientists so the best possible protein identifications can be realized.

## The Challenges of Protein Identification

The computational biologist is often confronted with data at a point in the research pipeline where many previous steps have been completed. For example, sample preparation, instrument choice, data acquisition, and peak picking will usually have been completed to produce the mass spectrum to be used for protein identification. These preliminary steps can be as important as the computational protein identification process itself. As an example of the importance of “upstream” steps in the proteomic process, up to 90% of tandem mass spectra in a typical liquid chromatography tandem mass spectrometry (LC-MS/MS) analysis cannot be identified using database search algorithms due to the poor quality of the spectra [12,14]. Spectra may be of poor quality for many reasons, including the presence of protein mixtures and contaminants, sample protein concentrations ranging over ten orders of magnitude [15], and incomplete digestion or fragmentation.

Once the data have been acquired by the mass spectrometer, there are features of these data that add further complexity and must be considered before a mass spectrum can produced. For example, the challenges involved in defining the center or presence of a peak [16–18] in turn define the sensitivity of peak detection and mass tolerance used for protein identification. Calibration issues [19–21] also influence the mass tolerance of spectral peaks. Such considerations are usually handled either automatically by the instrument software or by a mass spectrometry specialist. This information is then condensed into a tolerance parameter reported with the processed spectrum, which has a significant effect on protein identification. Likewise, digestion and fragmentation models used by protein identification packages can usually not be directly modified. However, the user should be aware that these models are far from perfect and are under active development [22,23].

The challenges of allowing for potentially poor, fragmented, or incomplete database annotation are, however, the responsibility of the user interpreting results returned from protein identification software. Studies show that for less well-characterized species, these choices can have significant effects on the interpretation of protein identification results [24,25].

The greatest challenges for both users and vendors of protein identification software revolve around the issue of simplicity versus versatility. The diversity in hardware and experimental parameters has forced software developers to produce products servicing a vast number of experimental and machine configurations, in many different laboratory environments. These software applications must also work well for the full range of subtleties existing between different biological experiments. Furthermore, the software must integrate into existing systems, and allow for changes in instrumentation while seamlessly integrating the most up-to-date information sourced from third parties, such as protein sequence databases [26].

This creates the design issue of how to allow enough flexibility to cover this diversity in hardware while incorporating all relevant knowledge about the biological question being asked. Inevitably, the user is heavily involved in the protein identification process by being forced to manually select many of the parameters for the search, a large proportion of which may not even be relevant to the particular experiment. This transfers much of the complexity of the task to the user, as evidenced by the length (around 90 pages!) of the typical basic user manual for a protein identification application. This highlights the need for both life scientists and computational biologists to understand the consequences their decisions at the searching stage will have on their ability to identify their proteins.

Finally, mass spectrometry data also raises issues of reporting and data management. Under what criteria are proteins “identified”? How should mass spectrometry related data be stored for later use and how are previous results affected by ever increasing database size? At present, there are few accepted standards for addressing such questions [27–30], although progress has been made [31–33]. Until such standards in data management and reporting are universal, it remains difficult to compare and interpret experiments. Some of the most pertinent challenges and solutions to these problems are covered in this review.

## Scoring Systems

The heart of all protein identification methods is the scoring system. Mass spectrometry data derived from the unidentified protein are compared with theoretical data from known proteins, and a score is assigned according to how well the two sets of data compare. Any score above an arbitrary confidence threshold is termed a “hit.” The top such hit is expected to identify the unknown protein. If there are no scores above this threshold (“no hits”), then the protein remains unidentified.

The development of scoring systems for protein identification began with the adaptation of well-developed general statistical methods common to many areas of science and engineering using methods such as cross correlation [34], Bayesian probability [35,36], expectation maximization [37], and machine learning [22,23], to name just a few. Progressively more sophisticated scoring systems have since been built by improving and combining standard scoring systems and by introducing novel statistical and search methods [38–40].

The limiting factor on all protein identification tools is the tradeoff between false positives and false negatives. It is absolutely essential to keep false positives to a minimum during protein identification because identifying the wrong protein can lead to a costly waste of time and resources. At the same time, it is clearly desirable to identify as many proteins as possible to draw maximum benefit from the experimental data. The ability of an algorithm to identify a protein is said to be its sensitivity, and its ability to distinguish true positives from false positives is said to be its specificity. As may be expected, there is a tradeoff between the two, embodied in a numerical threshold often called the confidence level, above which proteins are classed as identified. This is important for the researcher to bear in mind, since the balance between sensitivity and specificity will have a bearing on the threshold above which they are prepared to accept a protein as “identified.” For example, Chen et al. [41] report results for the popular peptide fragment fingerprinting (PFF) package called Mascot in a large cross-species study identifying human proteins in Escherichia coli databases using data collected on a high-performance LC-MS/MS LCQ ion trap mass spectrometer. They find correct proteins to have scores between 20 and 117, and incorrect proteins to have scores of up to 60. This demonstrates a fundamental property of protein identification software. As shown in Figure 1, the separation of true from false protein identifications based on a score is never perfect, and the general effectiveness of all protein identification algorithms should be viewed with this in mind.

**Figure 1:**
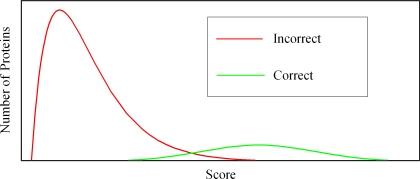
Score Distributions for Correct (Green) and Incorrect (Red) Protein Identifications Using a Scoring Scheme Note that there is no score threshold including all correct identifications while simultaneously excluding all false positives.

## Mass-Based Approaches

Using a mass-based approach, each protein in a database is theoretically subjected to the same experimental conditions as the protein to be identified. Typically, this will involve an enzymatic digestion and possible secondary fragmentation. This produces a theoretical mass spectrum (or spectra) for each protein in the database. These theoretical mass spectra are compared with the experimental spectrum. In theory, any method of comparison between two spectra can be a candidate for a scoring system, and in practice a variety of methods are used. Details of these methods are included later in this review. As an example, and possibly the most basic method for comparison, one can consider the shared peak count. The shared peak count, as the name implies, counts the number of peaks in the same position (shared) in both the experimental and theoretical spectra. The theoretical spectrum with the highest shared peak count is then said to be the closest match. Another widely used related scoring function is the “coverage.” Given each peak represents a fragment of the peptide, the coverage is the proportion of the protein covered by these matching fragments. Practical scoring systems use the combination of many such metrics to produce a final score.

### 

#### Peptide mass fingerprinting.

Peptide mass fingerprinting (PMF) was the first available method of protein identification using mass spectrometry, and is still widely used [5]. This method uses theoretical spectra each comprising the list of masses expected by an enzymatic digestion of each protein sequence in the reference database. The experimental spectrum consists of the masses of the digested protein fragments detected by the mass spectrometer such as the one shown in Figure 2.

**Figure 2:**
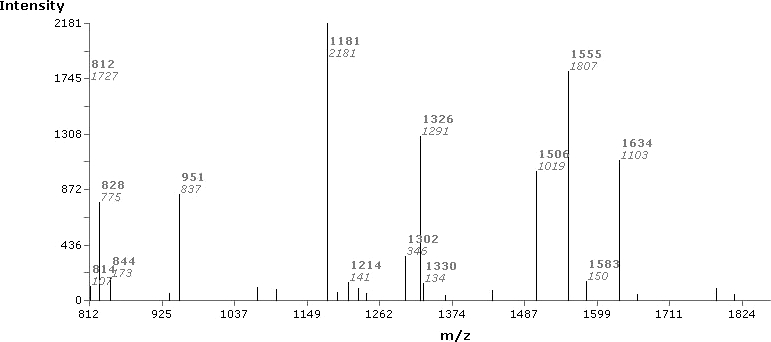
Peptide Mass Fingerprint Identified as Alpha-Synuclein in Mus musculus (IPI00115157) During the HUPO Brain Proteome Pilot Study The bold numbers associated with each peak are the m/z value, while the italic numbers associated with each peak show the intensity value. This “stick” spectrum has been processed from the raw output of the mass spectrometer. Available at http://www.ebi.ac.uk/pride/viewSpectrum.do?mzDataAccession=1681&spectrumReference=10021.

PMF is popular and works well in practice because it is relatively fast to compute PMF scores against a database. For good quality samples belonging to well-characterized model organisms, PMF can in many cases produce protein identifications with high confidence, especially in organisms with smaller genomes. Unfortunately, sometimes a sample spectrum does not resemble any theoretical spectra in the protein database closely enough to make a confident identification. This can happen for many reasons, such as unexpected post-translational or chemical modifications, splice variants, individual sequence variants (single nucleotide polymorphisms [SNPs], etc), or omissions and errors in the database. As more sophisticated methods for scoring PMF have been developed, more proteins can now be identified with confidence. This corresponds to a better separation of true from false positives using the scoring system. A wide variety of methods for attributing a score to the similarity between two spectra has been explored, with the most successful algorithms taking into consideration a number of factors to derive the final score. The next step involves deciding on a threshold: proteins whose scores are above this threshold are said to be identified. The definition of a threshold is difficult because setting a threshold too low will incorrectly identify a number of proteins (false positives), while setting the threshold too high may result in the correct protein not being identified (false negative). Statistical methods have been developed to determine the optimal threshold to keep the number of false positives below a given percentage [42,43]. The improvement of such PMF scoring systems is seeing diminishing returns, implying some fundamental limit to this approach. Nonetheless, recent innovation continues in PMF scoring [44,45] and applications [46], and PMF will likely remain an important tool for protein identification in the life sciences for some time.

One of the limitations of PMF is its sensitivity to database size. There is a direct effect on the statistical confidence a PMF algorithm can ascribe to protein identification as the search database grows. A larger database has an elevated chance of the experimental masses randomly matching theoretical peptide masses in these databases, thereby decreasing the confidence of protein identifications using PMF. Many experimentalists use PMF as a “first pass” to identify a protein, and if the identification fails or remains tentative, move on to methods such as PFF [35]. The most popular packages are, not surprisingly, the easiest to use, and include simple graphical interfaces, and return results along with a result a measure of confidence in the identification. Some of the more popular PMF packages are listed below in Table 1.

**Table 1:**
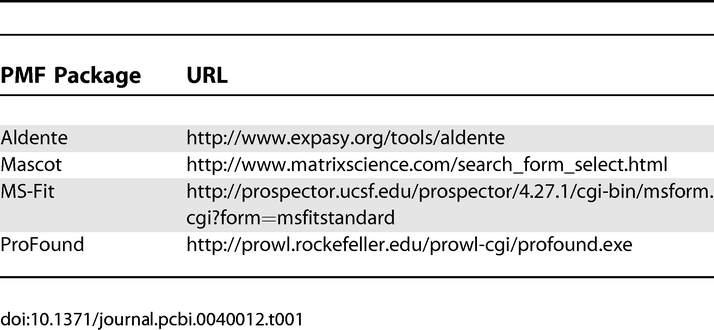
A Short List of Popular PMF Packages

Aldente [47] is hosted on the ExPASy Proteomics Server as one of a suite of bioinformatics tools. Released in 2004, Aldente uses a robust Hough transform to speed searches and find straight lines hidden in the data, making this tool more robust to noise than other PMF packages. A number of additional constraints can be input by the user, such as isoelectric point and molecular weight to restrict the effective database size. Unlike most other PMF packages, the user is able to select the parameters contributing to the final score and their proportions in order to “fine-tune” the search engine to a particular experiment. For example, a parameter exists for the likelihood of missed cleavages in the sample. Researchers confident of a complete digestion would select the corresponding parameter, allowing Aldente to produce a scoring scheme optimized for this experimental situation. The selection of some of these parameters is analogous to some of the inputs for other PMF packages, such as whether a modification is described as fixed or variable. Other such scoring parameters allow the user to introduce into the scoring function intensity information, modifications, and protein coverage, among other parameters and restrictions. The details of this tunable scoring scheme are available on the ExPASy Web site [48] along with supporting documentation. A threshold for identification is set after processing random sequences in the same parent mass range. The random sequence with the highest score becomes the threshold above which a protein is said to be identified.

Mascot [36] uses a proprietary scoring algorithm but is known to be based on the MOWSE algorithm [49], first described in 1993. By calculating the distribution of tryptic peptide lengths across the entire search database, a probability can be calculated for each observed peak for this match being purely random. Perkins et al. [36] describe in general terms the basis of this probabilistic scoring system, giving the user of this package an insight into how to interpret data generated via Mascot:

“The fundamental approach is to calculate the probability that the observed match between the experimental data set and each sequence database entry is a chance event. The match with the lowest probability is reported as the best match. Whether this match is also a significant match depends on the size of the database. To take a simple example, the calculated probability of matching six out of ten peptide masses to a particular sequence might be 10^−5^. This may sound like a promising result but, if the real database contains 10^6^ sequences, several scores of this magnitude may be expected by chance. A widely used significance threshold ..... is *p* < 0.05.

For a database of 10^6^ entries, this would mean that those with significant matches were those with probabilities of less than 5 × 10^−8^. .... we have adopted a convention often used in sequence similarity searches, and report a score which is −10Log_10_(*P*), where *P* is the probability. A significant match is typically a score of the order of 70.”

This means searches in smaller protein databases, such as bacterial databases, will generally have lower threshold scores for confidence than those conducted in larger databases for higher organisms. We can also infer that for noisy experimental spectra, for example those with contamination, these extra peaks contribute to the possibility of a random match, and thus raise the confidence score threshold for a given probability. Mascot automatically returns a score threshold with its results calculated to represent a confidence level of *p* < 0.05. Examples of the input data format and results format are available from the Matrix Science Web site [50].

MS-Fit [51] is also a probabilistic algorithm, again based on MOWSE, but runs over FASTA format [52] databases. MS-Fit has extended the basic MOWSE algorithm to include a number of additional options. These are detailed on the MS-Fit Web site [53]. MS-Fit first bins proteins according to the parent mass weight. Within each of these bins, a series of bins are created according to the tryptic peptide masses. This is done so that when calculating the probability of a random tryptic peptide match, it is calculated specifically for the distribution of these peptide masses for a given parent mass, effectively reducing the size of the search database. As we have seen in the extract from Pappin et al. above, this has the effect of lowering the threshold above which we can consider a protein as having been identified. MS-Fit also allows for the input of a number of possible contaminant masses. This allows the user to pre-filter any likely contaminants from the spectrum, thus increasing the quality of the spectrum against which a search is to be performed.

Profound [35] uses a Bayesian probability scoring system to score hits, using additional information outside of the set normally used by PMF algorithms, such as enzyme cleavage chemistry information, provisions for the knowledge that particular amino acids are present (or absent) in the sample protein, and previous experiments on the sample protein. Each piece of information functions as an additional constraint upon the search space of database proteins, therefore reducing the effective size of the database against which the search is conducted. As we have seen for the other PMF algorithms described above, a reduction of the effective size of the database has the effect of lowering the chance of a random match. Viewed in the Bayesian terms of Profound, this corresponds to a reduced probability of expected random matches in the more constrained search space, thus reducing the product component of the Bayesian equation [35], in turn increasing the overall probability for a correct match. Profound uses Gaussian distributed measurement errors in the probability calculations to more closely model real error, as opposed to the simple bounded “tolerance” error measurements used in other PMF algorithms. Profound has been extended to include a number of observed patterns in the probability calculation, such as the common occurrence of peptides with a common C- or N-terminal location when the experimental peptide masses are aligned against the sequence of the correct peptide, such as in the case of incomplete digestion. These “common ends” can be incorporated as corroborating evidence for a correct match. The same Bayesian framework can be readily extended to deal with mixtures of proteins by creating database entries that “fuse” combinations of single proteins, so that the probability of any small mixture (up to four proteins) can be given in absolute terms. There is no confidence level associated with a correct match because all Profound results are in the form of an absolute probability. It is left to the user to decide if the top-ranking candidate is sufficiently separate from other candidate peptides to declare the protein identified. Zhang et al. [35] suggest that the correct protein should have a probability very close to 1, while proteins in the same family or homologous proteins in another species will have probabilities in the order of 10^−10^, with unrelated proteins returning a probability of less than 10^−33^. The flexibility of a Bayesian approach has allowed Profound to remain competitive since its introduction in 2000.

A recent study by Chamrad et al. [6] using matrix-assisted laser desorption–ionization time-of-flight (MALDI-TOF) mass spectrometry data from a project mapping genes onto mouse chromosomes used expert interpretation of the spectra to identify 70% of the proteins, thus forming a reference set for PMF algorithm comparison. This study found the performance of Mascot and Profound to be similar, correctly identifying around 53% of proteins from the reference set at a 5% significance level, with MS-Fit identifying only 32% using the same input parameters. This study also looked at the effects of various parameters for Mascot and Profound queries. Profound performs better over the entire range of parameter settings including taxonomy restriction, mass accuracy variation, variable modifications, and missed cleavages. Mascot showed a slightly better performance only in the case where mass accuracy was better than 25 ppm. Overall, Profound identified slightly more proteins, showed a better separation between true and spurious identifications, and generated not a single random match above the 5% significance level throughout the experiment.

A study in yeast using 266 spectra gathered on MALDI-TOF instruments from three different manufacturers [7] found Mascot to outperform Profound, with Mascot identifying 45% of proteins while Profound identified 33% of the proteins. However, Mascot did give a single false positive identification, while Profound did not. This suggests that lifting the threshold for a Mascot identification to avoid all false positives, thus making the results comparable with the Profound result, would reduce the percentage of proteins identified using Mascot.

The claims for the highest rate of protein identifications belong to groups using consensus methods. These methods submit the query data independently to multiple search engines and combine the results. The rationale for this process is that marginal identifications may be corroborated or rejected by complementary packages. Experimentalists have been doing this independently for some time [8], usually in an ad hoc manner. However, recently, more rigorous statistical methods have been applied to the integration of the scores returned by each engine. One well-known example is ProteinScape [54]. This software is designed to accommodate a number of different proteomics workflows, including 2-D LC-MS/MS, LC–eletrospray ionization, and LC-MALDI. ProteinScape's consensus method claims an increase of identified proteins of up to 10% by taking a meta-score of Profound, Mascot, MS-Fit, and/or other algorithms. Details of the algorithm are not in the public domain, and the vendor provides only a short description of the meta-score as an “intelligent combination of scoring schemes.” Such consensus methods are now being adopted by large-scale projects [55], but are still not popular in smaller labs because these consensus programs are not free, and there are additional complexities in terms of running multiple PMF search engines.

Developments in mass spectrometry hardware, the explosive growth of database sizes, and computational advances necessitate the need for ever-higher sensitivity and accuracy in order to deliver gains in protein identification rates. These requirements have resulted in the emergence of a technique known as PFF, particularly in the high-throughput proteomics domain.

#### Peptide fragment fingerprinting.

Approaches using PFF data are the current mainstream of high-throughput protein identification. Proteins are first digested with an enzyme, and then individual peptides are selected to undergo further fragmentation to yield PFF spectra such as the one shown in Figure 3. The set of these spectra, along with information such as the parent mass of these fragmented peptides, are then used in the database search. There are many dozens of scoring systems described in the literature, but in most cases these consist of two steps: (1) attributing a score for each protein in the database and (2) calculating a measure of confidence that the top-ranking identified protein is not a false positive—such as in the case where the protein being investigated does not exist in the database.

**Figure 3:**
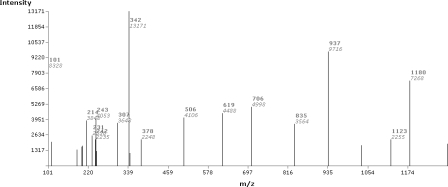
Example of a PFF spectrum from the HUPO Brain Proteome Project The bold numbers associated with each peak give the m/z value, while the italic numbers associated with the peak show the intensity value. This “stick” spectrum has been processed from the raw output of the mass spectrometer. Available at http://www.ebi.ac.uk/pride/viewSpectrum.do?mzDataAccession=1717&spectrumReference=32494.

PFF is the method of choice for high-throughput applications due to the additional information gained from secondary fragmentation. This information makes the protein identification process less sensitive to effects such as protein modifications and can generate higher statistical confidence in the correct identification than traditional PMF. Some of the more popular PFF packages are listed below in Table 2.

**Table 2:**
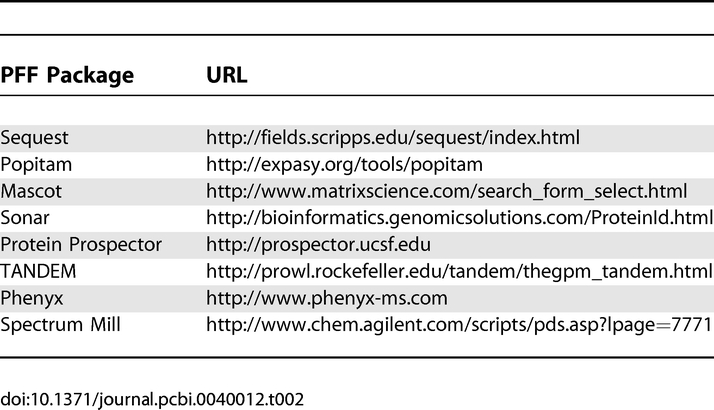
A List of Popular PFF Packages

Sequest and Mascot are arguably the two most popular packages for protein identification using PFF. Mascot is probabilistically based while also using some heuristics to improve scoring, but, like Spectrum Mill, ProteinProspector, and the most recent commercial PFF package, Phenyx, the details of the scoring process have not been published. Mascot, however, is known to be based on the probabilistic MOWSE algorithm [49], which uses the parent mass and the relative abundance of peptide masses for that parent mass as constraints on the search space. More than a decade has passed since the MOWSE algorithm was published, and Mascot now includes parameters not related to the features described in the original paper, such as selecting the type of mass spectrometer the input data comes from. It is therefore impossible to describe in any detail the process by which Mascot scores are generated, and a comparison with other engines can only be made empirically by analysis of the benchmarking papers discussed in this review.

Sequest uses a patented scoring algorithm utilising a cross-correlation approach. Figure 4 shows a simplified flowchart of the Sequest peptide identification process as described in U.S. patent 6,017,693. The preliminary closeness-of-fit *Sp* calculation can be determined by “a number of different scoring algorithms” [56], but the method described in the patent describes a scoring based on the length of a continuous y/b ion sequence interpreted from the input spectrum to produce a number of candidate sequences, to which a more sensitive and computationally intensive search can be applied to identify the correct protein in a later search of a candidate subset, assuming that the protein is both in the database and among the top-scoring candidate proteins. The correlation function produces scores based on the presence of significant peaks in the experimental spectrum corresponding to the expected y/b ion peaks in each theoretical spectrum in the database. Sequest outputs a ranked list of candidate peptide identifications, each reporting four scores.

**Figure 4:**
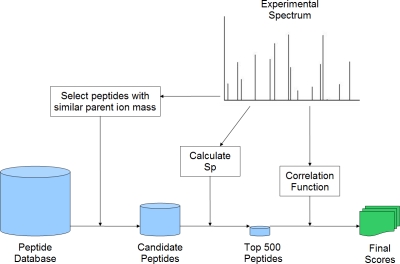
A Simplified Flowchart for the Sequest Algorithm Showing the Process by which Sequest Provides Scores Used to Identify Peptides Flowchart shows process as described in United States patent 6,017,693 [56]. Note that information from the mass spectrum is used three times: (1) as a filter to select only peptides from the database sharing a similar parent ion mass with the unknown peptide; (2) during a preliminary Sp “closeness-of-fit” filter to select the top 500 peptide candidates; and (3) through a correlation function to produce the final scores.

*Sp:* this is a preliminary score based on a computationally cheap increment/decrement schedule for identifying alignments between ions in the experimental and theoretical spectra. Sp is used as a first pass to gather a set of 500 peptides for further analysis. Larger peptides have a higher Sp. For example, a good hit for a 20-residue peptide will often have a Sp over 1,000, while a good hit for a 6-residue peptide will often be below 500.

*Cn:* the normalized correlation score is a measure of how well the theoretical y/b ion spectrum correlates to the experimental spectrum, corrected for peptide length. The uncorrected value is known as XCorr, the cross correlation. Cn has a range between 1 and 0 (normalized), with 1 being perfect correlation between experimental and theoretical spectra and a score of 0 indicative that there are no aligned peaks between the spectra.

*ΔCn:* this score gives a measure of how far the top score is above the other candidates. It is simply the difference between the top-ranking Cn and the Cn for each other peptide.

*Ions:* this value tells the user how many of the experimental ions align with theoretical ions. For example, 10/12 means that from the 12 peaks in the experimental input, 10 were matched in the theoretical peak list for that candidate peptide.

These measures provide important information for interpreting Sequest results. Obvious criteria for a confident identification are a high normalized correlation score (close to 1), a significant proportion of aligned ion peaks, and good separation from other candidate peptides. The distillation of Sequest results into identifications with corresponding confidence scores has been an area of active research [57–59], and led to the development of software such as PeptideProphet [60]. This well-known program aims to convert Sequest scores into probabilities so that some measure of confidence can be tied to a particular Sequest result. This is achieved by calculating for each high-ranking candidate peptide returned by Sequest a single “discriminant score” derived from a formula involving the various outputs of Sequest. The distribution of these discriminant scores can be approximated by Gaussian curves fitted to correct and incorrect areas of the distribution. Using these curves, probabilities for correct and incorrect identifications can then be generated for any new candidate identifications returned by Sequest.

A competing package, Popitam, was released in 2003 and uses a novel parsing of the MS/MS spectrum for matching against a database, followed by correlation scoring. This package has been specifically designed to address the limitations of other algorithms in the case of modifications or mutations in the source peptide [39]. The combinatorics usually involved in searches including such modifications is avoided by using search strategies based on Ant Colony Optimization algorithms, and borrows a representation used in de novo sequencing methods. Sonar [61] is capable of searching directly against genomic databases and is therefore able to identify proteins from unannotated genomes. TANDEM is an open source program deriving scores using expectation values for the observed and theoretical protein that are the same. Taxonomy files input into the system are optimized so that subsequent searches can be made faster than other PFF packages, and TANDEM claims the functionality of allowing for various protein point mutations during search.

A recent benchmarking paper produced by Kapp et al. [62] compared a number of the publicly available PFF packages listed above on a common dataset generated using electrospray ionization on an LCQ Deca XP IT (Thermo-Finnigan) from human samples used in the HUPO Plasma Proteome Project [63]. Different expert groups used their own preferred PFF software to identify proteins from the given dataset, either Sequest, Mascot, Sequest–PeptideProphet, Spectrum Mill, Sonar, or X!Tandem. The study found that, of the 608 proteins identified by at least one of the algorithms (union), 335 were identified by all of the algorithms (intersection), with 71 being identified by only a single algorithm. The proteins identified by only a single algorithm were then independently manually verified, with most being determined by this expert validation to be correct identifications. Sequest performed the best, identifying significantly more proteins at a fixed false positive rate of 1% and doing so with less sensitivity to various parameters such as charge state or cleavage agent. This study found Sequest and Spectrum Mill to be the most sensitive packages, and Mascot, Sonar, and X!Tandem to be better at distinguishing correct and incorrect peptide hits for the data acquired on the low-resolution electrospray ionization ion trap MS instrument.

Resing et al. [9] report similar results for shotgun analyses of the human erythroleukemia K562 cell line using a manually expert verified dataset. In this study, two popular packages, Mascot and Sequest, were each able to validate less than half of the potentially identifiable MS/MS spectra generated by an LCQ Classic IT MS (ThermoElectron). This study found for both Mascot and Sequest a 2-fold increase in the number of unique peptide assignments above the threshold when four replicate samples were subjected to analysis. There was considerable overlap between identified proteins, and manual verification determined that most of the proteins identified by only one of the algorithms were correct.

In 2004, Chamrad et al. [6] reported the results of a study exploring the selectivity and sensitivity of a number of popular PFF packages. A total of 89 mouse brain–derived proteins were separated using 2-D electrophoresis (2-DE), and digested in-gel before spectral acquisition on a REFLEX III MALDI-TOF MS (Bruker-Daltonik) using a SCOUT 384 source, in the context of a European mouse gene mapping project. A total of 70% of these proteins were identified using manual expert interpretation to form a reference set for evaluation of the PFF packages. Starting from a common parameter set, a total of 6,600 searches were performed to test the effects of various parameter settings. This study reports for Sequest a more obvious separation of correct identifications and random matches than Mascot, with Sequest identifying more than twice the number of proteins compared to Mascot based on Sequest's ΔCn score (threshold not specified) and Mascot's *p* < 0.05 threshold. Sequest was found to be less sensitive to parameter changes, including fragment and parent ion tolerances and especially variable modifications. Restricting the taxonomy of the search from “all” to “mouse” improved correct identifications for both engines by roughly 30%. The overall recommendations of the study include “a careful and sparing” use of variable modifications, restricting the taxonomy search as much as is appropriate, and searching with two missed cleavages as a good starting point if speed is not critical.

The results of these studies suggest: (1) there is a high level of overlap in the identified sets of proteins between PFF packages; (2) the thresholds used to exclude false positives also exclude up to half of the true positives; and (3) some algorithms were better at identifying certain types of proteins than others.

Despite the extensive literature on algorithm improvements, and the number of free implementations of these algorithms, many of these improvements are not being adopted by life scientists. In a review of protein identification packages, Shadforth [64] speculates that, although many improvements and alternatives exist to the most widely used packages, until these packages become more user-friendly and can be clearly shown to do better than the market leaders, they won't be adopted.

Although it is difficult to compare the various packages for protein identification, Kapp et al. [62] have published useful tables for a few of the more popular PFF engines so experimenters can determine the threshold corresponding to a particular false positive rate against the Human International Protein Index (IPI) database [65].

Meta scoring incorporating scores from multiple PFF packages has been well explored and successfully applied to significantly reduce the number of false positives, thereby allowing significantly more identifications for a given false positive rate [62,66–68]. While the above studies used ad hoc methods to combine the scores, programs exist which use more rigorous statistical methods to combine the scores of various packages. Such programs include MSPlus [9] which uses a heuristic set of rules applied to Mascot and Sequest results, followed by a least-squares fitting step between the two results to produce a sum-score for each candidate protein. Scaffold [69] also provides consensus and cross-validation features by probabilistically combining scores from Sequest, Mascot, and X!Tandem, and claims to significantly lower the false positive rate, thus allowing more identifications above a given confidence level.

## Tag-Based Approaches

Tag-based approaches begin with an attempt to extract peptide sequence information directly from the peptide fragmentation spectra. These methods are based on casting the problem into one of finding a maximum path length through a graph, a problem already known to have efficient solutions, and are based on a seminal paper by Dancik et al. [70]. The process of inferring protein sequence from MS/MS data is known as de novo sequencing. Due to the high complexity of most MS/MS spectra, de novo sequencing tools often return short, ambiguous sequences known as “tags.” These tags are then searched against a database. Although many of these tags may randomly align with sections of protein sequence right across the genome, the correct protein identification is expected to have multiple alignments with sequence tags derived from the unknown protein. Tag-based approaches have been successfully used to identify proteins from larger EST databases that are more inclusive than curated databases. They have also been used for finding homologous proteins in other species [71], an area where mass-based approaches, and particularly PMF, have been shown to have limited applicability [72].

Not surprisingly, tag-based approaches appeared as the first de novo sequencing methods were becoming available [73]. A number of popular packages available for de novo sequence interpretation and subsequent tag-based searching are listed below in Table 3.

**Table 3:**
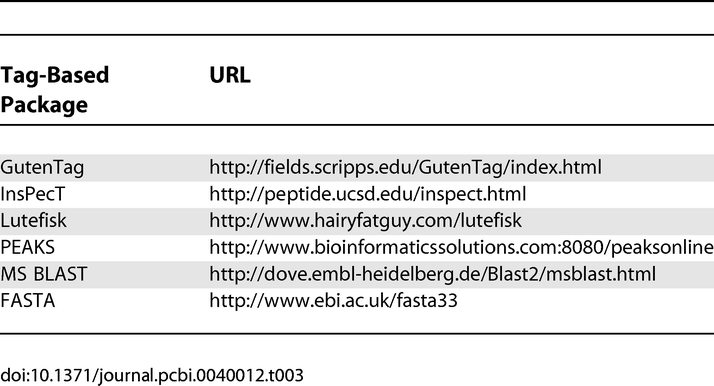
Popular Packages for De Novo Sequencing of MS Data and Tag-Based Protein Identification Engines

GutenTag [74] is a popular tag-based package released in 2003 by the same group responsible for the popular PFF package Sequest, and is available free for nonprofit organizations. Lutefisk is available as source code in C, allowing the experimenter to tailor aspects of the scoring function or any other aspect of reporting and calculation. It works by first identifying “significant” ions, followed by the collection of evidence for N- and C-terminal ions from the spectra. A list of candidate sequences is generated for passing onto a tag-based program for alignment of these candidate sequences with proteins in a database. InsPecT [75] is a recently introduced tag-based package based on a probability model for assessing the accuracy of candidate sequence tags. PEAKS is a proprietary package, and as such has not published details of its implementation. MSBLAST and FASTA do not infer de novo sequence but are popular alignment programs evolved from DNA alignment roots.

### 

#### De novo sequencing.

PEAKS is the current standard for de novo sequencing tasks prior to submission to an alignment program for protein identification. It reports results with a confidence measure and has been shown to outperform the popular Lutefisk as well as various software packages from instrument manufacturers [76]. For quadrupole TOF (QTOF) data across a range of spectrum qualities, the authors claim 41% perfectly correct sequences and 94% of sequences to have six consecutive correctly sequenced amino acids.

De novo sequencing quality is highly dependent on the precision of the mass spectrometer and the quality of the spectra. Advances in hardware accuracy and precision have a great effect on the ability of de novo algorithms to correctly and accurately infer longer stretches of protein sequence. Quality spectra as well as high precision greatly constrain the possible sequences capable of generating the observed spectrum. Thus, the short list of possible peptides, to be later submitted to a tag-based search, may contain longer and therefore more specific sequences, resulting in more confident identifications.

Preparatory methods to improve the quality of spectra intended for de novo sequencing is an active area of research [77–80]. For researchers using de novo techniques and who have input into the sample preparation stage, we recommend the survey of such techniques because the extended length of de novo interpreted sequences under these conditions may be greatly increased, thus allowing significantly more confident protein identification. A good starting point would be the recent review by Joss et al. [81].

#### Tag-based search algorithms.

Most current tag-based methods use a basic adaptation of the BLAST [82] or FASTA [83] algorithms. These are already in common use in the life sciences for gene and protein sequence alignments. For use in tag-based searching, the algorithms are modified for the much shorter peptide sequences usually generated by MS/MS, typically in the order of eight to 15 amino acids, and to handle the errors and ambiguities resulting from the alternate possible sequence interpretations when de novo sequencing [84]. The tag-based algorithms listed above have shown similar performance for average length [85] and for the slightly shorter than normal sequences resulting from poorer sequence quality [86]. Recent computational advances improving the quality of de novo interpretation of MS/MS sequence information have made tag-based approaches competitive with PFF methods in terms of specificity and sensitivity [10–11,74,87,88], and the number of tag-based algorithms for protein identification is rapidly growing and gaining popularity. This has prompted current market-leading PFF software packages such as Mascot to include tag-search capability in their packages.

Tag-based approaches are much faster than PFF searches; Tanner et al. [75] report a two order of magnitude speed-up over the commonly used Sequest through using their tag-based method InsPecT, as well as demonstrating a much better scalability when scaled to include added modifications or protein mixtures. The speed-up is due to a more efficient and sensitive use of tags to exclude the vast bulk of potential protein matches considered in a first pass, although the authors note that performance for single protein identifications is not better than the PFF package X!Tandem in terms of speed and sensitivity, as this package has already incorporated a similar filtering system.

Tag-based methods have been designed to function in environments where exact matches are not expected—for example, searching against databases of other species—and as such have different methods for determining the statistical significance of a result under these conditions. This translates to a much higher sensitivity in these types of applications when compared with other methods such as the PFF methods already discussed [8,89]. Tag-based methods are therefore the method of choice for searching error-prone EST databases and cross-species protein identification due to their natural handling of imperfect sequence alignment [71].

A study by Habermann et al. [87] of cross-species tag-based searches using theoretically generated peptide sequences randomly mutated to mimic the limited accuracy to de novo methods showed that very few hits will be missed by this type of search once the sequence similarity between species exceeds 60%, even when identification is based on as few as five peptides of ten amino acids in length. Furthermore, near-optimal performance was reached by searching for a protein with 15 query peptides of tentenmino acids per peptide, with acceptable performance in related species with as few as three query peptides of ten amino acids in length. An earlier study by Cordwell et al. [8] on cross-species protein identification found similar results and also showed tag-based methods to be superior over PFF for this type of application. However, obtaining 10–15 spectra of sufficient quality for accurate de novo sequencing is technically demanding and not always possible, especially from low-end machines. These studies nonetheless show the current utility and theoretical promise of tag-based approaches for unknown protein identification using databases of both known and related species.

#### Other available packages.

There is a great number and variety of protein identification packages other than those listed in this review. Many of these packages have been tailored to provide identifications for particular classes of proteins, or even glycans [45], or use certain techniques and report superior performance to established general protein identification engines listed in this review for their specific application. A quick survey of other available tools and packages in many cases can turn up software ideal for a particular application. Brief descriptions and intended uses of all the packages listed in Tables 1–3 and others can be found in a review by Shadforth et al. [64]. An exhaustive list of protein identification tools can be found at http://www.molecularstation.com/bioinformatics/link/Proteomics/Protein_Identification_Tools and http://www.proteomesoftware.com/Proteome_software_link_software.html.

## Protein Identification Comparisons and Reporting

Much effort has been made by various groups to devise metrics allowing the various protein identification packages to be compared. Such metrics include: (1) calculating expectation values for the number of hits expected for a given score [28–30]; (2) the hit-ratio (i.e., the ratio of the peaks submitted in the experimental spectrum matched in the theoretical spectrum); and (3) sequence coverage (i.e., the proportion of the protein sequence covered by the peptides matched between experimental and theoretical spectra) [27].

However, these metrics are not regularly used in the literature, and there is still no firm consensus on how the results of protein identifications can be reported and compared. Recognizing the critical need to clearly compare experiments, some proteomics journals have already introduced standards for protein identification reporting [32,33], and others are likely to follow. The metrics required to be presented along with protein identifications for these publishers include but are not limited to: (1) supporting information detailing the use of all processing steps, experimental design, scoring methods used, software and database versions, and all parameters used in the search; (2) sequence coverage and/or hit rate; (3) measures of certainty such as *p*-values; (4) justifying evidence for identifications made on single peptides, for a particular protein within a protein family, or proteins identified in another species; and (5) multiple replicates for complex analyses.

The problem of standardizing mass spectrometry–related data formats and vocabulary is being addressed by the HUPO Proteomics Standards Initiative [31]. This group has released a standard format for encapsulation of peak list data (mzData) and has an alpha version of the successor to this format under development. Known as mzML, this format will merge the competing mzXML and mzData formats. Details of the new format can be found on the mzML development page [90]. The same group is also developing a format known as AnalysisXML for the encapsulation of parameters and results from protein identifications. These formats are enjoying increasing support from instrument manufactures and software vendors, and are rapidly being adopted up by the proteomics community.

## Discussion

A consistent message found in this review of protein identification algorithms is that the best results for protein identification are extracted through the use of consensus programs used to collect the results from various packages and distill their results. This is particularly the case in the mass-based approaches of PMF and PFF. Through such methods, the strengths of some packages can be exploited, while weaknesses in others are mitigated. The only difficulty with this approach is the added expense and difficulty of operating multiple-search algorithms as well as the consensus-scoring software for compiling and analyzing the different results. However, the added performance gained, in terms of correct protein identification, would appear to encourage the investment in using consensus-based methods.

All of the methods require the use, at some point, of a reference sequence database for identifying the proteins expressed in the sample. This presents extra challenges for researchers working with less well-characterized species. In this scenario, tag-based methods are preferred because of the reduced computational complexity of searching for diverged proteins. Mass-based methods require matching of peptide or peptide fragment masses to their theoretical equivalents derived from a sequence database. A single amino acid change, with the exception of a change between leucine and isoleucine, will change the mass of the peptide or peptide fragment with a resultant effect on the ability of the algorithm to correctly identify the protein. In contrast, with tag-based methods, particularly if the tag-matching process is tolerant of sequence variation, sequence changes have less of an impact on the ability to correctly match database entries. Thus, cross-species databases can be more effectively used to aid in protein identification.

Identifying the single best package for each application from the available literature is at present extremely difficult due to a number of factors. Each package claims advantages over a number of others. These claims are often backed up with compelling results. While some of the comparative studies cited above have produced work of excellent scope and quality, many of these results show marginal differences between packages or show contradictory results to other studies. Furthermore, across all the studies, only a small fraction of the available packages have been considered and evaluated. Similarly contradictory results are reported not only in comparisons between various packages, but also between approaches, such as between mass-based and tag-based approaches. This indicates the datasets and thresholds used in such comparisons have a critical importance on the outcome of such experiments, and that the high variability in machine and experimental setups complicates analysis. The state of data standards and lack of benchmarks therefore makes it difficult to make an effective comparison, implying the need for sustained directed research on the creation of suitable benchmarks. While the increasing availability of data in public repositories and tightening standards will no doubt ameliorate the problem, until this basic benchmarking problem is overcome, no single package or approach can conclusively be declared to outperform all others, expect, perhaps, in the specific circumstances used in particular studies. For such benchmarking work to be successful, it is important that it be broad, replicable, and routine, because each software package is constantly evolving, so a benchmark can at best produce a comparison likely to quickly become redundant as newer versions of packages are released. This, in turn, points to the need for an ongoing process of benchmark-based testing, in which new algorithms and techniques, or developments in existing packages, are regularly re-evaluated to measure performance and provide guidance to life-science researchers seeking to extract the most from their proteomic experiments.

It is clear there is much room for improvement in protein identification techniques as, despite the many advances in the field, it is still evident that fewer than half of the proteins in a typical proteomics study can be identified [6–11,87]. Progress is continually being made to increase the separation between true and false positives for each of the available approaches (PMF, PFF, and tag-based methods). These advances are likely to be incorporated into the major packages as their utility is demonstrated, but are unlikely to be independently adopted by the average life scientist due to their complexity [64].

The uptake of new standards for reporting and particularly for data formats holds the promise of direct comparison between experiments, and more importantly, a solid suite of benchmarks against which new methods and techniques can be measured. These standards are being eagerly adopted by the computational biology community and will serve to streamline development efforts.

At present, the greatest focus in improving protein identification software is on the following: (1) developing better scoring metrics or including additional information [91–94]; (2) improving fragmentation models. The inclusion of new metrics [95] and use of new techniques [23] applied to fragmentation modeling allows for better prediction of theoretical spectra. This, in turn, leads to more discriminating scoring systems; (3) data representations for clustering or filtering to improve speed and efficiency [12,96,97].

These methods can massively speed searches by reducing the size of the database being searched through the use of statistical methods to cheaply reject the majority of non-matching database entries, or by improving the speed at which comparisons can be made.

The above, purely algorithmic, improvements complement new techniques for sample preparation and data acquisition such as chemical derivatization [77,81] as well as orthogonal fragmentation techniques [98,99]. Such techniques provide more, cleaner, or corroborating information upon which protein identification algorithms can operate and underlie the most significant recent advances in the ability to identify proteins.

Protein identification software faces the ongoing challenge of incorporating these diverse advances in hardware, software, and methodology as we move toward the ability to rapidly and confidently identify a greater proportion of proteins from every experiment. 

## Abbreviations:

LC: liquid chromatography
MALDI-TOF: matrix-assisted laser desorption–ionization time-of-flight
MS/MS: tandem mass spectrometry
PFF: peptide fragment fingerprinting
PMF: peptide mass fingerprinting
